# Hexa-μ_2_-benzoato-bis­(2,2′-bipyrid­yl)trimanganese(II) monohydrate

**DOI:** 10.1107/S1600536808012518

**Published:** 2008-05-03

**Authors:** Hong-Chang Yao, Ning Wang, Li Zhang, Zhong-Jun Li

**Affiliations:** a Department of Chemistry, Zhengzhou University, Zhengzhou 450001, People’s Republic of China; b Department of Chemistry and Chemical Engineering, Pingdingshan Institute of Technology, Pingdingshan 467044, People’s Republic of China; c School of Biological and Chemical Engineering, Nangyang Institute of Technology, Nangyang 473004, People’s Republic of China

## Abstract

The complex molecule of the title compound, [Mn_3_(C_7_H_5_O_2_)_6_(C_10_H_8_N_2_)_2_]·H_2_O, contains a linear array of divalent manganese ions. The central Mn^II^ atom, which is located on a crystallographic inversion center, is coordinated octa­hedrally by six benzoate O atoms. The two terminal Mn^II^ ions are six-coordinated by four benzoate O atoms and two N atoms of 2,2′-bipyridyl. The central Mn^II^ atom and the terminal Mn^II^ ions are bridged by four benzoate ligands in a bidentate fashion, whereas the other two carboxyl­ate ligands form bridges through one O atom only and chelate the terminal Mn^II^ atom. The mol­ecules pack together *via* van der Waals attractions and C—H⋯O hydrogen bonds.

## Related literature

For general background, see: Mukhopadhyay *et al.* (2002 ▶) and references therein; Gatteschi *et al.* (2003 ▶) and references therein; Yao *et al.* (2006 ▶); Ma *et al.* (2007 ▶). For related literature, see: Desiraju *et al.* (2002 ▶) and references therein. For related structures, see: Ménage *et al.* (1991 ▶); Tangoulis *et al.* (1996 ▶); Fernández *et al.* (2002 ▶).
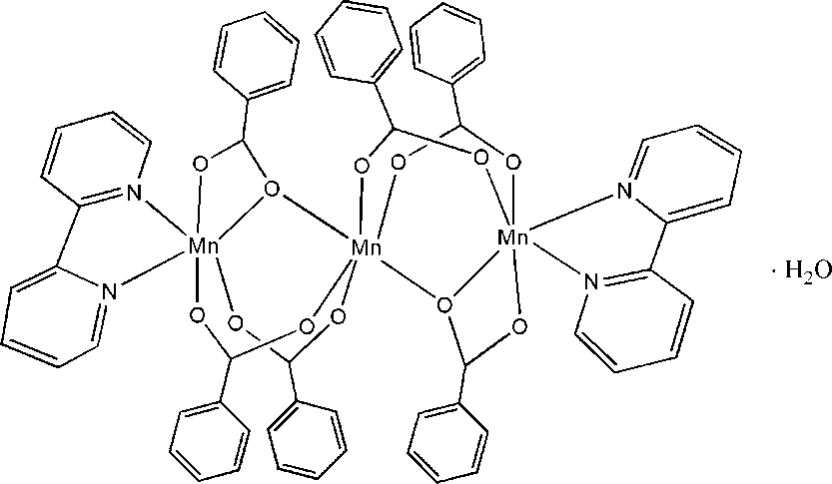


## Experimental

### 

#### Crystal data

[Mn_3_(C_7_H_5_O_2_)_6_(C_10_H_8_N_2_)_2_]·H_2_O
*M* *_r_* = 1221.86
Triclinic, 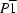

*a* = 11.2312 (5) Å
*b* = 11.7544 (2) Å
*c* = 11.994 (3) Å
α = 72.046 (3)°
β = 71.094 (1)°
γ = 80.418 (2)°
*V* = 1421.1 (4) Å^3^
*Z* = 1
Mo *K*α radiation
μ = 0.72 mm^−1^
*T* = 291 (2) K
0.30 × 0.20 × 0.10 mm

#### Data collection

Bruker SMART APEX CCD diffractometer
Absorption correction: multi-scan (*SADABS*; Bruker, 2000 ▶) *T*_min_ = 0.841, *T*_max_ = 0.932
11367 measured reflections
5553 independent reflections
4007 reflections with *I* > 2σ(*I*)
*R*_int_ = 0.036

#### Refinement

*R*[*F* ^2^ > 2σ(*F* ^2^)] = 0.060
*wR*(*F* ^2^) = 0.138
*S* = 1.08
5553 reflections
382 parameters
H atoms treated by a mixture of independent and constrained refinement
Δρ_max_ = 0.29 e Å^−3^
Δρ_min_ = −0.70 e Å^−3^

### 

Data collection: *SMART* (Bruker, 2000 ▶); cell refinement: *SAINT* (Bruker, 2000 ▶); data reduction: *SAINT*; program(s) used to solve structure: *SHELXTL* (Sheldrick, 2008 ▶); program(s) used to refine structure: *SHELXTL*; molecular graphics: *SHELXTL*; software used to prepare material for publication: *SHELXTL*.

## Supplementary Material

Crystal structure: contains datablocks I, global. DOI: 10.1107/S1600536808012518/rk2087sup1.cif

Structure factors: contains datablocks I. DOI: 10.1107/S1600536808012518/rk2087Isup2.hkl

Additional supplementary materials:  crystallographic information; 3D view; checkCIF report

## Figures and Tables

**Table 1:** Hydrogen-bond geometry (Å, °)

*D*—H⋯*A*	*D*—H	H⋯*A*	*D*⋯*A*	*D*—H⋯*A*
C6—H6⋯O3^i^	0.93	2.56	3.458 (5)	161
C9—H9⋯O2^ii^	0.93	2.53	3.287 (5)	139
C22—H22⋯O4	0.93	2.55	3.149 (5)	122

Symmetry codes: (i) 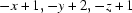; (ii) 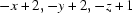.

**Fig. 1.**
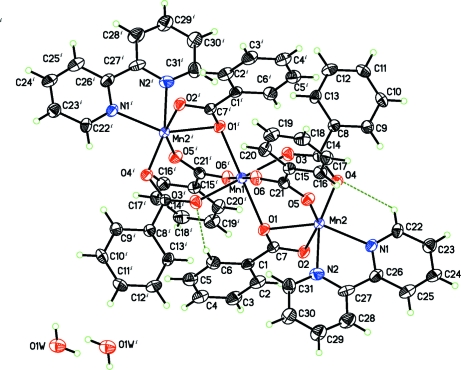
Perspective view of the title compound, with the atom numbering scheme. The displacement ellipsoids are drawn at the 30% probability level. The H atoms are presented as a small spheres of arbitrary radius. The intramolecular H–bonds are drawn as dashed lines. Symmetry codes: (i) 1-x, 2-y, 1-z.

**Fig. 2.**
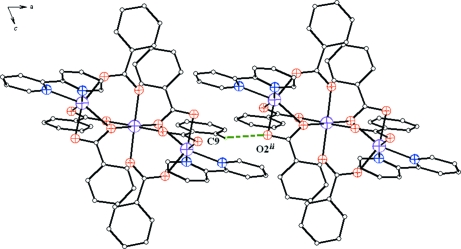
The intermolecular C—H···O hydrogen bond between the molecule units. H atoms not included in the hydrogen bond were omitted for clarify. Symmetry codes: (ii) 2-x, 2-y, 1-z.

## supplementary crystallographic information

### Comment

The chemistry of manganese in various states and various nuclearities has received much attention since the discovery of their potential as models for the oxygen–evolving complex of photosystem II (Mukhopadhyay *et al.*, 2002 and references therein) and as single–domain nanoscale magnetic particles (Gatteschi *et al.*, 2003 and references therein). We have previously reported the crystal structure of manganese complexes with benzoate, phosphonate and 2,2'–bipyridyl ligands (Yao *et al.*, 2006; Ma *et al.*, 2007). In this paper, we report the crystal structure of a Mn_3_–complex with benzoate and 2,2'–bipyridyl ligands.

The structure of the title compound is composed of a linear array of trimanganese ions and a water molecule (Fig. 1). The linear complex includes a central, octahedral Mn^II^ ion [Mn1] that is located on a crystallographic inversion center. Its coordination sphere is composed of six oxygen atoms from six different benzoates. The central Mn1 ion is flanked by two octahedrally distorted Mn^II^ ion [Mn2]. For the Mn2 atom, four of its six coordination positions are occupied by the oxygen atoms O1, O2, O4 and O5 from the benzoate ligands. The remaining two positions are filled with the nitrogen atoms from the 2,2'–bipyridyl ligand. The Mn—N bond lengths are 2.259 (3)Å and 2.271 (3)Å. The Mn—O bond lengths are in the range of 2.075 (2)–2.300 (2) Å. The bond lengths and angles may be compared with the corresponding values in similar complexe of Mn^II^: Mn_3_(*Ac*O)_6_(*bipy*)_2_ (Ménage *et al.*, 1991), Mn_3_(*Ac*O)_6_(*pybim*)_2_ (Tangoulis *et al.*, 1996) and [Mn_3_(µ–ClCH_2_COO)_6_(*bipy*)_2_] (Fernández *et al.*, 2002). The Mn1 and Mn2 atoms are bridged by six benzoate ligands, four of which show the common µ_2_–η^1^:η^1^ coordination mode while the other two adopt the µ_3_–η^2^:η^1^ coordination mode (Scheme).

The oxidation state assignments for Mn ions as Mn^II^ are based on the following observations. The trinuclear molecules is neutral and is composed of six monoanionic benzoate donors (*e.g.* benzoates) and two neutral 2,2'–bipyridyl ligands. Additional support for this oxidation level is provided by the almost equal distances of the Mn—O and Mn—N bond lengths, which is typical for a *d*^5^ system (Ménage *et al.*, 1991; Fernαndez *et al.*, 2002).

The hydrogen–bond parameters of the title compound are listed in Table. The molecule shows C—H···O intramolecular H bond with C···O distance of 3.149 (5)Å and 3.458 (5)Å and C—H···O angle of > 115° (dashed line in Fig. 1). The distance and angle values are typical for these types of hydrogen bonds (Desiraju *et al.*, 2002 and references therein). The title compound packed together *via* van der Waals attractions as well as intermolecular C9—H9···O2^ii^ hydrogen bonds (dashed line in Fig. 2). Symmetry codes: (ii) 2-*x*, 2-*y*, 1-*z*.

### Experimental

[Mn_3_O(*Ph*CO_2_)_6_(*py*)_2_^.^(H_2_O)] (0.1079 g, 0.01 mmol) (where is *py* = pyridine) was dissolved in 10 ml CH_3_CN, to which a solution of 1,1,1–tris(hydroxymethyl)methylamine (0.0121 g, 0.01 mmol) and 2,2'–bipyridyl (0.0156 g, 0.01 mmol) in dichloromethane (10 ml) was added. After stirring at room temperature for half an hour, the solution was filtered and the filtrate was allowed to evaporate slowly in air. A small crop of the dark–brown X–ray quality crystals of the title compound was formed in several days. Anal. Calc. for C_62_H_46_Mn_3_N_4_O_12_^.^H_2_O (C_62_H_48_Mn_3_N_4_O_13_): C 60.94%, H 3.96%, N 4.59%. Found: C 60.69%, H 3.83%, N 4.68%. FT–IR (KBr pellet, cm^-1^): 3440 br, 3062 w, 2361 w, 1610s, 1570 s, 1492 w, 1470 w, 1446 w, 1391 s, 1173 w, 1067 w, 1025 w, 836 w, 768 w, 718 m, 674 w, 640 w, 463 w. Thermogravimetric analysis of the title compound shows one step weight loss in the temperature range 323–473 K. The total weight loss (1.8%) is slightly higher than the calculated value of 1.5% for the removal of one free water molecule.

### Refinement

All H atoms attached to C atoms were fixed geometrically and treated as riding with C—H = 0.93 Å and *U*_iso_(H) = 1.2*U*_eq_(C). H atoms of the water molecule were located in difference Fourier maps and included in the subsequent refinement using restraint (O—H = 0.85 (1) Å) with *U*_iso_(H) = 1.2*U*_eq_(O). In the last stage of refinement, it was treated as riding on the O atom. The free water molecule in the crystal lattice was treated as disorder with 50% occupation rate.

### Crystal data

**Table:**

[Mn_3_(C_7_H_5_O_2_)_6_(C_10_H_8_N_2_)_2_]·H_2_O	*Z* = 1
*M**_r_* = 1221.86	*F*_000_ = 627
Triclinic, *P*1	*D*_x_ = 1.428 Mg m^−^^3^
Hall symbol: -P 1	Mo *K*α radiation λ = 0.71073 Å
*a* = 11.2312 (5) Å	Cell parameters from 4735 reflections
*b* = 11.7544 (2) Å	θ = 2.1–26.5º
*c* = 11.994 (3) Å	µ = 0.73 mm^−^^1^
α = 72.046 (3)º	*T* = 291 (2) K
β = 71.0940 (10)º	Block, dark–brown
γ = 80.418 (2)º	0.30 × 0.20 × 0.10 mm
*V* = 1421.1 (4) Å^3^	

### Data collection

**Table:**

Bruker SMART APEX CCD diffractometer	5553 independent reflections
Radiation source: Sealed tube	4007 reflections with *I* > 2σ(*I*)
Monochromator: Ggraphite	*R*_int_ = 0.036
*T* = 291(2) K	θ_max_ = 26.0º
φ and ω scans	θ_min_ = 2.5º
Absorption correction: multi-scan(SADABS; Bruker, 2000)	*h* = −13→13
*T*_min_ = 0.841, *T*_max_ = 0.932	*k* = −14→14
11367 measured reflections	*l* = −14→14

### Refinement

**Table:**

Refinement on *F*^2^	Secondary atom site location: difference Fourier map
Least-squares matrix: Full	Hydrogen site location: inferred from neighbouring sites
*R*[*F*^2^ > 2σ(*F*^2^)] = 0.060	H atoms treated by a mixture of independent and constrained refinement
*wR*(*F*^2^) = 0.138	*w* = 1/[σ^2^(*F*_o_^2^) + (0.07*P*)^2^] where *P* = (*F*_o_^2^ + 2*F*_c_^2^)/3
*S* = 1.08	(Δ/σ)_max_ < 0.001
5553 reflections	Δρ_max_ = 0.29 e Å^−^^3^
382 parameters	Δρ_min_ = −0.70 e Å^−^^3^
Primary atom site location: structure-invariant direct methods	Extinction correction: none

### Special details

**Table:**

Geometry. All s.u.'s (except the s.u. in the dihedral angle between two l.s. planes) are estimated using the full covariance matrix. The cell s.u.'s are taken into account individually in the estimation of s.u.'s in distances, angles and torsion angles; correlations between s.u.'s in cell parameters are only used when they are defined by crystal symmetry. An approximate (isotropic) treatment of cell s.u.'s is used for estimating s.u.'s involving l.s. planes.
Refinement. Refinement of *F*^2^ against ALL reflections. The weighted *R*–factor *wR* and goodness of fit *S* are based on *F*^2^, conventional *R*–factors *R* are based on *F*, with *F* set to zero for negative *F*^2^. The threshold expression of *F*^2^ > σ(*F*^2^) is used only for calculating *R*–factors(gt) *etc*. and is not relevant to the choice of reflections for refinement. *R*–factors based on *F*^2^ are statistically about twice as large as those based on *F*, and *R*–factors based on ALL data will be even larger.

### Fractional atomic coordinates and isotropic or equivalent isotropic displacement parameters (Å^2^)

**Table:**

	*x*	*y*	*z*	*U*_iso_*/*U*_eq_	Occ. (<1)
C1	0.7698 (3)	0.7996 (3)	0.3087 (3)	0.0385 (8)	
C2	0.8860 (4)	0.7842 (4)	0.2269 (4)	0.0525 (10)	
H2	0.9590	0.7944	0.2423	0.063*	
C3	0.8943 (4)	0.7532 (4)	0.1208 (4)	0.0589 (11)	
H3	0.9724	0.7449	0.0644	0.071*	
C4	0.7860 (4)	0.7353 (4)	0.1005 (4)	0.0572 (10)	
H4	0.7914	0.7139	0.0304	0.069*	
C5	0.6702 (4)	0.7485 (4)	0.1825 (4)	0.0546 (10)	
H5	0.5980	0.7351	0.1678	0.066*	
C6	0.6594 (4)	0.7815 (3)	0.2865 (4)	0.0496 (9)	
H6	0.5805	0.7916	0.3411	0.060*	
C7	0.7607 (3)	0.8311 (3)	0.4229 (3)	0.0369 (7)	
C8	0.7418 (3)	1.2525 (3)	0.5247 (3)	0.0364 (7)	
C9	0.8542 (3)	1.2741 (3)	0.5377 (4)	0.0454 (9)	
H9	0.9084	1.2099	0.5641	0.054*	
C10	0.8856 (4)	1.3894 (4)	0.5120 (4)	0.0489 (9)	
H10	0.9613	1.4029	0.5199	0.059*	
C11	0.8053 (4)	1.4850 (3)	0.4745 (4)	0.0503 (9)	
H11	0.8251	1.5630	0.4598	0.060*	
C12	0.6939 (4)	1.4641 (3)	0.4587 (4)	0.0519 (10)	
H12	0.6410	1.5284	0.4301	0.062*	
C13	0.6621 (3)	1.3488 (3)	0.4851 (3)	0.0401 (8)	
H13	0.5867	1.3355	0.4763	0.048*	
C14	0.7122 (3)	1.1261 (3)	0.5471 (3)	0.0312 (6)	
C15	0.3814 (3)	0.9013 (3)	0.9078 (3)	0.0361 (7)	
C16	0.4312 (4)	0.8754 (3)	1.0053 (3)	0.0448 (8)	
H16	0.5171	0.8538	0.9926	0.054*	
C17	0.3569 (4)	0.8806 (3)	1.1204 (3)	0.0471 (9)	
H17	0.3920	0.8627	1.1847	0.056*	
C18	0.2299 (4)	0.9127 (4)	1.1389 (3)	0.0513 (10)	
H18	0.1788	0.9167	1.2163	0.062*	
C19	0.1785 (4)	0.9388 (4)	1.0444 (3)	0.0513 (10)	
H19	0.0926	0.9603	1.0576	0.062*	
C20	0.2541 (3)	0.9332 (3)	0.9284 (3)	0.0450 (8)	
H20	0.2185	0.9511	0.8644	0.054*	
C21	0.4675 (3)	0.9023 (3)	0.7814 (3)	0.0355 (7)	
C22	0.9965 (3)	0.8848 (4)	0.6997 (3)	0.0449 (8)	
H22	0.9783	0.9660	0.6664	0.054*	
C23	1.1066 (4)	0.8490 (4)	0.7370 (4)	0.0521 (9)	
H23	1.1606	0.9051	0.7286	0.063*	
C24	1.1315 (4)	0.7329 (4)	0.7846 (4)	0.0543 (10)	
H24	1.2029	0.7072	0.8114	0.065*	
C25	1.0518 (4)	0.6497 (4)	0.7947 (4)	0.0577 (11)	
H25	1.0705	0.5682	0.8264	0.069*	
C26	0.9434 (4)	0.6893 (4)	0.7568 (4)	0.0491 (9)	
C27	0.8531 (4)	0.6079 (3)	0.7629 (4)	0.0534 (10)	
C28	0.8616 (5)	0.4857 (4)	0.8167 (4)	0.0660 (12)	
H28	0.9259	0.4506	0.8522	0.079*	
C29	0.7743 (4)	0.4173 (4)	0.8172 (4)	0.0615 (12)	
H29	0.7790	0.3352	0.8540	0.074*	
C30	0.6809 (4)	0.4675 (4)	0.7648 (4)	0.0605 (11)	
H30	0.6216	0.4210	0.7649	0.073*	
C31	0.6762 (4)	0.5914 (4)	0.7106 (4)	0.0562 (10)	
H31	0.6119	0.6273	0.6754	0.067*	
Mn1	0.5000	1.0000	0.5000	0.03159 (18)	
Mn2	0.74951 (5)	0.86247 (4)	0.63436 (5)	0.03183 (15)	
N1	0.9164 (3)	0.8058 (3)	0.7104 (3)	0.0423 (7)	
N2	0.7615 (3)	0.6596 (3)	0.7081 (3)	0.0508 (8)	
O1	0.6536 (2)	0.8502 (2)	0.4961 (2)	0.0391 (5)	
O2	0.8604 (2)	0.8344 (2)	0.4472 (2)	0.0439 (6)	
O3	0.6136 (2)	1.1124 (2)	0.5272 (2)	0.0458 (6)	
O4	0.7876 (2)	1.0433 (2)	0.5852 (2)	0.0423 (6)	
O5	0.5826 (2)	0.8747 (2)	0.7718 (2)	0.0441 (6)	
O6	0.4186 (2)	0.9293 (2)	0.6956 (2)	0.0419 (6)	
O1W	0.4239 (7)	0.4880 (6)	0.9786 (6)	0.0601 (16)	0.50
H1A	0.477 (11)	0.463 (9)	1.019 (11)	0.072*	0.50
H1B	0.369 (10)	0.538 (9)	1.009 (9)	0.072*	0.50

### Atomic displacement parameters (Å^2^)

**Table:**

	*U*^11^	*U*^22^	*U*^33^	*U*^12^	*U*^13^	*U*^23^
C1	0.0372 (17)	0.0400 (18)	0.0360 (18)	0.0098 (15)	−0.0094 (14)	−0.0151 (15)
C2	0.049 (2)	0.050 (2)	0.046 (2)	0.0097 (18)	−0.0083 (17)	−0.0080 (18)
C3	0.058 (3)	0.064 (3)	0.048 (2)	0.016 (2)	−0.015 (2)	−0.018 (2)
C4	0.057 (2)	0.052 (2)	0.055 (2)	0.0120 (19)	−0.015 (2)	−0.013 (2)
C5	0.058 (2)	0.051 (2)	0.053 (2)	0.0051 (19)	−0.015 (2)	−0.0171 (19)
C6	0.051 (2)	0.050 (2)	0.050 (2)	−0.0006 (18)	−0.0131 (18)	−0.0185 (18)
C7	0.0314 (17)	0.0371 (17)	0.0416 (18)	0.0034 (13)	−0.0111 (14)	−0.0123 (15)
C8	0.0300 (16)	0.0370 (17)	0.0381 (17)	−0.0062 (14)	−0.0056 (13)	−0.0073 (14)
C9	0.0373 (19)	0.043 (2)	0.057 (2)	−0.0079 (16)	−0.0130 (17)	−0.0125 (17)
C10	0.051 (2)	0.052 (2)	0.053 (2)	−0.0175 (18)	−0.0137 (18)	−0.0209 (18)
C11	0.058 (2)	0.037 (2)	0.058 (2)	−0.0135 (18)	−0.0113 (19)	−0.0169 (17)
C12	0.053 (2)	0.037 (2)	0.054 (2)	0.0064 (18)	−0.0139 (19)	−0.0031 (17)
C13	0.045 (2)	0.0393 (18)	0.0344 (18)	−0.0028 (15)	−0.0203 (15)	0.0019 (14)
C14	0.0278 (15)	0.0362 (16)	0.0293 (16)	−0.0020 (13)	−0.0063 (12)	−0.0109 (13)
C15	0.0365 (18)	0.0329 (16)	0.0335 (16)	−0.0069 (14)	−0.0056 (13)	−0.0040 (13)
C16	0.046 (2)	0.051 (2)	0.0355 (18)	0.0003 (17)	−0.0087 (16)	−0.0147 (16)
C17	0.057 (2)	0.051 (2)	0.0338 (18)	−0.0042 (18)	−0.0148 (17)	−0.0104 (16)
C18	0.057 (2)	0.052 (2)	0.0324 (19)	0.0003 (19)	0.0024 (17)	−0.0137 (17)
C19	0.046 (2)	0.055 (2)	0.038 (2)	0.0038 (18)	−0.0014 (16)	−0.0083 (17)
C20	0.0410 (19)	0.054 (2)	0.0337 (19)	−0.0038 (16)	−0.0095 (15)	−0.0038 (16)
C21	0.0381 (18)	0.0377 (17)	0.0316 (17)	−0.0053 (14)	−0.0087 (14)	−0.0109 (13)
C22	0.0337 (18)	0.058 (2)	0.045 (2)	−0.0045 (16)	−0.0126 (16)	−0.0150 (18)
C23	0.044 (2)	0.055 (2)	0.055 (2)	−0.0048 (18)	−0.0130 (18)	−0.0110 (19)
C24	0.049 (2)	0.055 (2)	0.051 (2)	0.0158 (19)	−0.0130 (18)	−0.0155 (19)
C25	0.050 (2)	0.056 (2)	0.052 (2)	0.014 (2)	−0.0111 (19)	−0.0068 (19)
C26	0.046 (2)	0.055 (2)	0.043 (2)	0.0117 (18)	−0.0175 (17)	−0.0133 (18)
C27	0.053 (2)	0.038 (2)	0.057 (2)	0.0160 (18)	−0.0121 (19)	−0.0095 (18)
C28	0.066 (3)	0.048 (2)	0.069 (3)	−0.004 (2)	−0.023 (2)	0.007 (2)
C29	0.061 (3)	0.049 (2)	0.059 (3)	−0.011 (2)	−0.020 (2)	0.013 (2)
C30	0.052 (2)	0.051 (2)	0.067 (3)	−0.017 (2)	−0.018 (2)	0.006 (2)
C31	0.059 (3)	0.054 (2)	0.056 (3)	−0.014 (2)	−0.026 (2)	−0.002 (2)
Mn1	0.0309 (4)	0.0296 (4)	0.0336 (4)	−0.0016 (3)	−0.0084 (3)	−0.0091 (3)
Mn2	0.0282 (3)	0.0330 (3)	0.0353 (3)	0.00136 (19)	−0.0123 (2)	−0.0091 (2)
N1	0.0362 (16)	0.0501 (18)	0.0422 (16)	0.0069 (13)	−0.0157 (13)	−0.0155 (14)
N2	0.0525 (19)	0.0386 (17)	0.057 (2)	−0.0001 (15)	−0.0145 (16)	−0.0105 (15)
O1	0.0311 (12)	0.0478 (14)	0.0392 (13)	0.0059 (10)	−0.0112 (10)	−0.0167 (11)
O2	0.0318 (12)	0.0555 (15)	0.0466 (14)	0.0069 (11)	−0.0138 (11)	−0.0200 (12)
O3	0.0367 (13)	0.0517 (15)	0.0519 (15)	−0.0090 (11)	−0.0094 (11)	−0.0190 (12)
O4	0.0375 (13)	0.0357 (13)	0.0546 (16)	−0.0019 (10)	−0.0179 (12)	−0.0095 (11)
O5	0.0388 (14)	0.0503 (15)	0.0379 (13)	0.0044 (11)	−0.0074 (10)	−0.0124 (11)
O6	0.0432 (13)	0.0556 (15)	0.0260 (12)	−0.0107 (11)	−0.0074 (10)	−0.0091 (11)
O1W	0.060 (4)	0.049 (3)	0.064 (4)	−0.005 (3)	−0.009 (3)	−0.015 (3)

### Geometric parameters (Å, °)

**Table:**

C1—C2	1.378 (5)	C19—H19	0.9300
C1—C6	1.411 (5)	C20—H20	0.9300
C1—C7	1.494 (5)	C21—O6	1.251 (4)
C2—C3	1.400 (6)	C21—O5	1.256 (4)
C2—H2	0.9300	C22—N1	1.348 (5)
C3—C4	1.375 (6)	C22—C23	1.406 (5)
C3—H3	0.9300	C22—H22	0.9300
C4—C5	1.372 (6)	C23—C24	1.327 (6)
C4—H4	0.9300	C23—H23	0.9300
C5—C6	1.380 (6)	C24—C25	1.386 (6)
C5—H5	0.9300	C24—H24	0.9300
C6—H6	0.9300	C25—C26	1.395 (6)
C7—O2	1.254 (4)	C25—H25	0.9300
C7—O1	1.271 (4)	C26—N1	1.334 (5)
C7—Mn2	2.634 (4)	C26—C27	1.478 (6)
C8—C13	1.384 (5)	C27—N2	1.358 (5)
C8—C9	1.394 (5)	C27—C28	1.382 (6)
C8—C14	1.499 (5)	C28—C29	1.365 (7)
C9—C10	1.374 (5)	C28—H28	0.9300
C9—H9	0.9300	C29—C30	1.355 (6)
C10—C11	1.375 (6)	C29—H29	0.9300
C10—H10	0.9300	C30—C31	1.400 (6)
C11—C12	1.396 (6)	C30—H30	0.9300
C11—H11	0.9300	C31—N2	1.334 (5)
C12—C13	1.374 (5)	C31—H31	0.9300
C12—H12	0.9300	Mn1—O3^i^	2.139 (2)
C13—H13	0.9300	Mn1—O3	2.139 (2)
C14—O3	1.251 (4)	Mn1—O6^i^	2.166 (2)
C14—O4	1.254 (4)	Mn1—O6	2.166 (2)
C15—C20	1.377 (5)	Mn1—O1	2.251 (2)
C15—C16	1.385 (5)	Mn1—O1^i^	2.251 (2)
C15—C21	1.509 (5)	Mn2—O5	2.075 (2)
C16—C17	1.377 (5)	Mn2—O4	2.101 (2)
C16—H16	0.9300	Mn2—N1	2.259 (3)
C17—C18	1.376 (6)	Mn2—N2	2.271 (3)
C17—H17	0.9300	Mn2—O2	2.282 (3)
C18—C19	1.366 (6)	Mn2—O1	2.300 (2)
C18—H18	0.9300	O1W—H1A	0.85 (13)
C19—C20	1.390 (5)	O1W—H1B	0.85 (10)
			
C2—C1—C6	119.8 (3)	C25—C24—H24	119.7
C2—C1—C7	120.3 (3)	C24—C25—C26	119.3 (4)
C6—C1—C7	119.9 (3)	C24—C25—H25	120.4
C1—C2—C3	120.1 (4)	C26—C25—H25	120.4
C1—C2—H2	120.0	N1—C26—C25	120.8 (4)
C3—C2—H2	120.0	N1—C26—C27	115.7 (3)
C4—C3—C2	119.5 (4)	C25—C26—C27	123.5 (4)
C4—C3—H3	120.2	N2—C27—C28	120.9 (4)
C2—C3—H3	120.2	N2—C27—C26	115.9 (3)
C5—C4—C3	120.7 (4)	C28—C27—C26	123.2 (4)
C5—C4—H4	119.6	C29—C28—C27	119.2 (4)
C3—C4—H4	119.6	C29—C28—H28	120.4
C4—C5—C6	120.8 (4)	C27—C28—H28	120.4
C4—C5—H5	119.6	C30—C29—C28	120.8 (4)
C6—C5—H5	119.6	C30—C29—H29	119.6
C5—C6—C1	119.0 (4)	C28—C29—H29	119.6
C5—C6—H6	120.5	C29—C30—C31	118.2 (4)
C1—C6—H6	120.5	C29—C30—H30	120.9
O2—C7—O1	120.7 (3)	C31—C30—H30	120.9
O2—C7—C1	118.8 (3)	N2—C31—C30	121.8 (4)
O1—C7—C1	120.5 (3)	N2—C31—H31	119.1
O2—C7—Mn2	60.01 (18)	C30—C31—H31	119.1
O1—C7—Mn2	60.82 (17)	O3^i^—Mn1—O3	180.0
C1—C7—Mn2	174.0 (2)	O3^i^—Mn1—O6^i^	91.63 (10)
C13—C8—C9	119.1 (3)	O3—Mn1—O6^i^	88.37 (10)
C13—C8—C14	121.0 (3)	O3^i^—Mn1—O6	88.37 (10)
C9—C8—C14	119.8 (3)	O3—Mn1—O6	91.63 (10)
C10—C9—C8	120.6 (4)	O6^i^—Mn1—O6	180.000 (1)
C10—C9—H9	119.7	O3^i^—Mn1—O1	87.97 (9)
C8—C9—H9	119.7	O3—Mn1—O1	92.03 (9)
C9—C10—C11	120.2 (4)	O6^i^—Mn1—O1	88.49 (9)
C9—C10—H10	119.9	O6—Mn1—O1	91.51 (9)
C11—C10—H10	119.9	O3^i^—Mn1—O1^i^	92.03 (9)
C10—C11—C12	119.6 (3)	O3—Mn1—O1^i^	87.97 (9)
C10—C11—H11	120.2	O6^i^—Mn1—O1^i^	91.51 (9)
C12—C11—H11	120.2	O6—Mn1—O1^i^	88.49 (9)
C13—C12—C11	120.3 (3)	O1—Mn1—O1^i^	180.000 (1)
C13—C12—H12	119.9	O5—Mn2—O4	96.34 (10)
C11—C12—H12	119.9	O5—Mn2—N1	111.32 (11)
C12—C13—C8	120.3 (3)	O4—Mn2—N1	90.61 (11)
C12—C13—H13	119.9	O5—Mn2—N2	90.34 (11)
C8—C13—H13	119.9	O4—Mn2—N2	162.30 (11)
O3—C14—O4	125.6 (3)	N1—Mn2—N2	71.69 (12)
O3—C14—C8	116.9 (3)	O5—Mn2—O2	152.16 (10)
O4—C14—C8	117.5 (3)	O4—Mn2—O2	94.63 (10)
C20—C15—C16	118.3 (3)	N1—Mn2—O2	94.03 (10)
C20—C15—C21	121.6 (3)	N2—Mn2—O2	86.83 (11)
C16—C15—C21	120.0 (3)	O5—Mn2—O1	95.16 (9)
C17—C16—C15	121.7 (4)	O4—Mn2—O1	105.17 (9)
C17—C16—H16	119.2	N1—Mn2—O1	147.59 (9)
C15—C16—H16	119.2	N2—Mn2—O1	90.47 (11)
C18—C17—C16	119.1 (4)	O2—Mn2—O1	57.22 (8)
C18—C17—H17	120.4	O5—Mn2—C7	123.82 (10)
C16—C17—H17	120.4	O4—Mn2—C7	102.37 (11)
C19—C18—C17	120.3 (4)	N1—Mn2—C7	120.78 (10)
C19—C18—H18	119.8	N2—Mn2—C7	87.25 (12)
C17—C18—H18	119.8	O2—Mn2—C7	28.41 (9)
C18—C19—C20	120.3 (4)	O1—Mn2—C7	28.86 (9)
C18—C19—H19	119.8	C26—N1—C22	118.7 (3)
C20—C19—H19	119.8	C26—N1—Mn2	118.9 (3)
C15—C20—C19	120.3 (4)	C22—N1—Mn2	122.1 (2)
C15—C20—H20	119.9	C31—N2—C27	119.1 (4)
C19—C20—H20	119.9	C31—N2—Mn2	123.1 (3)
O6—C21—O5	125.6 (3)	C27—N2—Mn2	117.4 (3)
O6—C21—C15	117.7 (3)	C7—O1—Mn1	136.2 (2)
O5—C21—C15	116.7 (3)	C7—O1—Mn2	90.3 (2)
N1—C22—C23	122.5 (4)	Mn1—O1—Mn2	104.10 (9)
N1—C22—H22	118.8	C7—O2—Mn2	91.6 (2)
C23—C22—H22	118.8	C14—O3—Mn1	149.7 (2)
C24—C23—C22	118.2 (4)	C14—O4—Mn2	121.3 (2)
C24—C23—H23	120.9	C21—O5—Mn2	138.0 (2)
C22—C23—H23	120.9	C21—O6—Mn1	130.7 (2)
C23—C24—C25	120.5 (4)	H1A—O1W—H1B	109 (10)
C23—C24—H24	119.7		

Symmetry codes: (i) −*x*+1, −*y*+2, −*z*+1.

### Hydrogen-bond geometry (Å, °)

**Table:**

*D*—H···*A*	*D*—H	H···*A*	*D*···*A*	*D*—H···*A*
C6—H6···O3^i^	0.93	2.56	3.458 (5)	161
C9—H9···O2^ii^	0.93	2.53	3.287 (5)	139
C22—H22···O4	0.93	2.55	3.149 (5)	122

Symmetry codes: (i) −*x*+1, −*y*+2, −*z*+1; (ii) −*x*+2, −*y*+2, −*z*+1.
